# Development of a Viral-Like Particle Candidate Vaccine Against Novel Variant Infectious Bursal Disease Virus

**DOI:** 10.3390/vaccines9020142

**Published:** 2021-02-10

**Authors:** Yulong Wang, Nan Jiang, Linjin Fan, Li Gao, Kai Li, Yulong Gao, Xinxin Niu, Wenying Zhang, Hongyu Cui, Aijing Liu, Qing Pan, Changjun Liu, Yanping Zhang, Xiaomei Wang, Xiaole Qi

**Affiliations:** 1 State Key Laboratory of Veterinary Biotechnology, Harbin Veterinary Research Institute, Avian Immunosuppressive Diseases Division, The Chinese Academy of Agricultural Sciences, Harbin 150069, China; xyylong@126.com (Y.W.); jiangnan3596@163.com (N.J.); 18346080676@163.com (L.F.); gaoli@caas.cn (L.G.); likai@caas.cn (K.L.); gaoyulong@caas.cn (Y.G.); Nqct17@163.com (X.N.); unstoppable0402@outlook.com (W.Z.); cuihongyu@caas.cn (H.C.); Liuaijing@caas.cn (A.L.); panqing@caas.cn (Q.P.); liuchangjun@caas.cn (C.L.); zhangyanping@caas.cn (Y.Z.); 2 OIE Reference Laboratory for Infectious Bursal Disease, Harbin Veterinary Research Institute, The Chinese Academy of Agricultural Sciences, Harbin 150069, China; 3 Jiangsu Co-innovation Centre for Prevention and Control of Important Animal Infectious Disease and Zoonosis, Yangzhou University, Yangzhou 225009, China

**Keywords:** novel variant infectious bursal disease virus, vaccine, viral-like particle

## Abstract

Infectious bursal disease (IBD), an immunosuppressive disease of young chickens, is caused by infectious bursal disease virus (IBDV). Novel variant IBDV (nVarIBDV), a virus that can evade immune protection against very virulent IBDV (vvIBDV), is becoming a threat to the poultry industry. Therefore, nVarIBDV-specific vaccine is much needed for nVarIBDV control. In this study, the VP2 protein of SHG19 (a representative strain of nVarIBDV) was successfully expressed using an *Escherichia coli* expression system and further purified via ammonium sulfate precipitation and size-exclusion chromatography. The purified protein SHG19-VP2-466 could self-assemble into 25-nm virus-like particle (VLP). Subsequently, the immunogenicity and protective effect of the SHG19-VLP vaccine were evaluated using animal experiments, which indicated that the SHG19-VLP vaccine elicited neutralization antibodies and provided 100% protection against the nVarIBDV. Furthermore, the protective efficacy of the SHG19-VLP vaccine against the vvIBDV was evaluated. Although the SHG19-VLP vaccine induced a comparatively lower vvIBDV-specific neutralization antibody titer, it provided good protection against the lethal vvIBDV. In summary, the SHG19-VLP candidate vaccine could provide complete immune protection against the homologous nVarIBDV as well as the heterologous vvIBDV. This study is of significance to the comprehensive prevention and control of the recent atypical IBD epidemic.

## 1. Introduction

Infectious bursal disease (IBD) caused by infectious bursal disease virus (IBDV) is an important immunosuppressive disease, responsible for enormous economic losses in the poultry industry worldwide [1]. IBDV is a non-enveloped virus, and a member of *Avibirnavirus* genus of the family *birnaviridae*. The genome of IBDV consists of two segments of double-stranded RNA (segments A and B) [1,2]. The segment A encodes a non-structural protein VP5 [3] and a polyprotein (pVP2-VP4-VP3), which can be further self-cleaved into the precursor of VP2 (pVP2), VP4, and VP3 [4]. During the process of IBDV maturation, the mature VP2 (441 residues) (amino acid [aa] 1-441) can be generated from the self-cleavage of pVP2 (aa 1-512), where its C-terminal (aa 442-512) of pVP2 end can be truncated [5,6]. VP2, the main IBDV host-protective antigen [7] that possesses a series of neutralizing epitopes [8,9], is an important determinant for virulence, antigenic variation, and cell tropism of IBDV [10,11]. Segment B encodes VP1, an RNA-dependent RNA polymerase, which is responsible for the genome transcription and translation of IBDV [12,13,14,15].

In 1957, classic IBDV was first reported in the USA [16]. Variant IBDV, which can break through the immune protection against classic IBDV, was described in 1987 [17]. Subsequently, very virulent IBDV (vvIBDV) with a high acute mortality has caused severe economic losses worldwide [18,19]. With reasonable immunization procedures and improved feeding management, vvIBDV is gradually being controlled. Since 2017, a novel variant IBDV (nVarIBDV), which is genetically different from the early variant IBDV, has become widely prevalent in immunized chicken farms in China [20]. Although the nVarIBDV cannot cause death of chickens, it directly damages immune organs, leading to immunosuppression, and seriously affects the weight gain and production performance of the infected flocks [20,21,22,23]. Recently, an epidemic of nVarIBDV has appeared in Japan and has brought negative economic impact [23].

Vaccination is the optimal strategy for controlling IBD [24]. However, almost all current commercial vaccines target vvIBDV, and these vaccines cannot sufficiently protect vaccinated chickens from nVarIBDV [22,25]. Therefore, it is important to develop antigenicity matching vaccines for the comprehensive prevention and control of nVarIBDV. In this study, a viral-like particle (VLP) candidate vaccine of nVarIBDV was developed using prokaryotic expression system, and its potential to be a vaccine candidate was evaluated in vitro and in vivo.

## 2. Materials and Methods

### 2.1. Viruses and Cells

The novel variant IBDV representative strain SHG19 [20] was previously isolated and identified by the Avian Immunosuppressive Disease Division, Harbin Veterinary Research Institute (HVRI), Chinese Academy of Agricultural Sciences (CAAS) (hereinafter referred to as “our lab”). The Chinese vvIBDV representative strain HLJ0504 [26] was also previously identified by our lab. The cell-adapted reassortment IBDV, rGtVarVP2 [27] or rGtHLJVP2 [28], which expresses the major protective antigen VP2 of SHG19 or HLJ0504 strain on the backbone of attenuated strain of IBDV, was previously rescued by our lab. Both rGtVarVP2 and rGtHLJVP2 can adapt to DF1 cells and induce cytopathic effect (CPE). DF1 cells were cultured in Dulbecco’s modified Eagle’s medium (DMEM) (Sigma-Aldrich, St. Louis, MO, USA) at 37 °C in a humidified 5% CO2 incubator.

### 2.2. Animals

Specific-pathogen-free (SPF) white Leghorn chickens were obtained from the Experimental Animal Center of HVRI, CAAS. The chickens were kept in negative-pressure-filtered air isolators. Animal experiments were performed in accordance with the animal ethics guidelines and approved protocols of HVRI of CAAS (ethical approval number SYXK (Hei) 2017-009).

### 2.3. Construction of Recombinant Expression Plasmid

**Figure 1:**
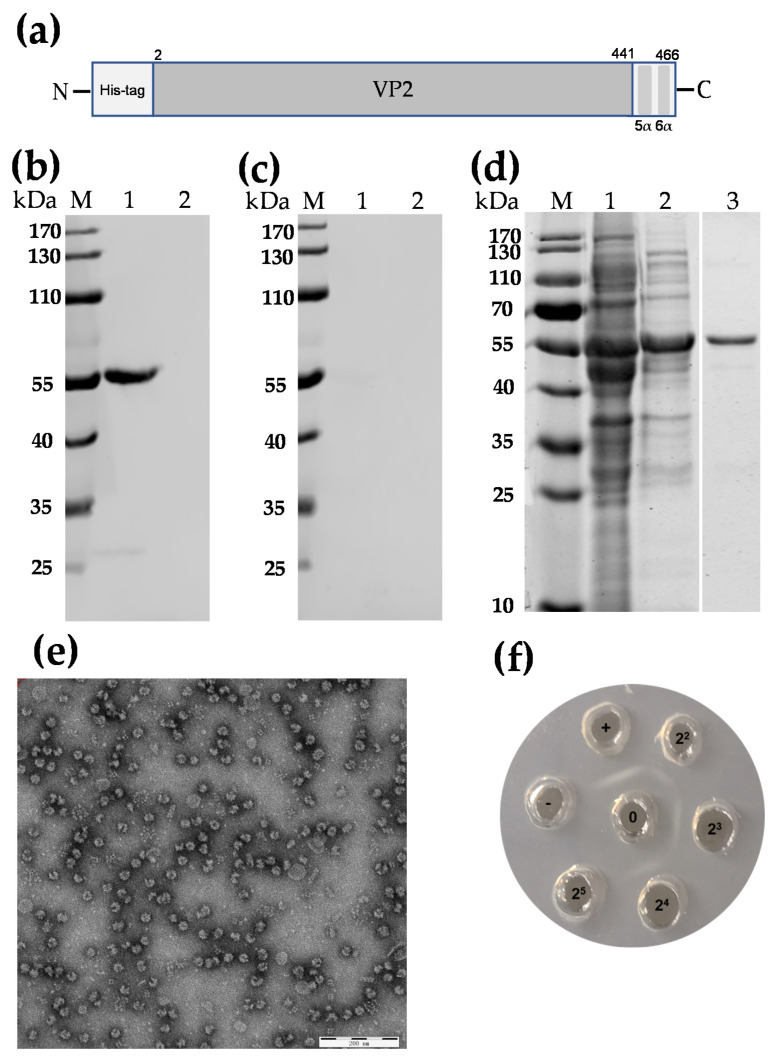
Preparation and identification of SHG19-VLP. (**a**) The Schematic diagram of the recombinant protein SHG19-VP2-466 (not drawn to scale). The SHG19-VP2-466 protein is a truncated pVP2 (amino acids [aa] 2-466) fusing with a N-terminal His-tag containing an initiation codon (ATG); VP2: aa 2-441 of pVP2; 5α: 5α-helix (aa 443-452 of pVP2); 6α: 6α-helix (aa 456-462 of pVP2); (**b**) Western blotting of the fusion protein SHG19-VP2-466 with an anti-VP2 monoclonal antibody (7D4). Lane M: Marker; Lane 1: the SHG19-VP2-466 in the supernatant of the pCo-HHT28-SHGVP2-466-transformed *E. coli*; Lane 2: supernatant of the pCold Ⅰ-transformed *E. coli*; (**c**) Western blotting of the fusion protein SHG19-VP2-466 with an anti-Flag monoclonal antibody. Lane M: Marker; Lane 1: the SHG19-VP2-466 in the supernatant of the pCo-HHT28-SHGVP2-466-transformed *E. coli*; Lane 2: supernatant of the pCold Ⅰ-transformed *E. coli*; (**d**) SDS-PAGE analysis of the fusion protein SHG19-VP2-466. Lane M: Marker; Lane 1: the fusion protein SHG19-VP2-466 protein sample; Lane 2: the purification of the fusion protein SHG19-VP2-466 via ammonium sulfate precipitation; Lane 3: the purification product of the fusion protein SHG19-VP2-466 via size-exclusion chromatography; (**e**) TEM image of the purified protein SHG19-VP2-466 (named SHG19-VLP). (**f**) AGP analysis of SHG19-VLP. 0: Antiserum; +: antigen of IBDV; -: PBS as a control; 2^2^–2^5^: dilution ratio of SHG19-VLP.

A partial pVP2 gene of SHG19 strain (nt 4-1398) with a His-tag gene [29] in the N-terminal (Figure 1a) was subcloned into a pCold-I vector (Takara, China) previously digested with *Kpn*I and *Eco*RI. The recombinant plasmid was transformed into competent *Escherichia coli* (*E. coli*) DH5α for selection. To confirm that the coding sequence was in-frame, the recombinant plasmid named pCo-HHT28-SHGVP2-466 was purified and sequenced by Comate Biosciences Company (Changchun, China).

### 2.4. Expression and Purification of SHG19-VP2-466

The recombinant expression vector pCo-HHT28-SHGVP2-466 was transformed into *E. coli* Transetta (DE3) for expressing the fusion protein named SHG19-VP2-466. The selected positive clone was inoculated in 4 mL LB medium with 100 μg ampicillin/mL at 220 rpm for 12 h in 37 °C incubator. Then, 2 mL of the bacteria culture was added into a 500 mL shaker flask containing 200 mL fresh LB medium with 100 μg/mL ampicillin, and cultured at 220 rpm in a 37 °C incubator. When the OD600 value reached 0.6, the culture was supplemented with 20 mM Isopropyl β-D-1-thiogalactopyranoside (IPTG) for inducing the expression of the VP2 fusion protein, followed by shock culture at 180 rpm for 22 h at 22 °C. Cells were harvested and centrifuged at 8000× *g* for 15 min at 4 °C, the supernatant was discarded and the pellet was resuspended by 20 mL buffer A (20 mM phosphate, pH 6.5). The resuspended sample were further disintegrated by sonication. After centrifugation at 10,000× *g* for 20 min at 4 °C, the supernatant was stored at 4 °C until use.

The protein sample was first purified via ammonium sulfate precipitation. A saturated ammonium sulfate solution was slowly added to the protein sample at a 1:1 volume ratio. After stirring continuously with a magnetic stirrer for 5 min, the mixture centrifuged at 10,000× *g* for 10 min at 25 °C. Subsequently, the supernatant was removed and the sediment was completely resuspended in buffer A. The soluble target protein was recovered by centrifugation (10,000× *g*, 10 min) at 25 °C. After passing through 0.45-μm filter, the filtrate was subjected to size-exclusion chromatography using Sepharose 6 Fast Flow column (2.6 cm in diameter and 90 cm in length, GE Healthcare, Boston, MA, USA). Briefly, the protein sample was loaded onto a pre-equilibrated chromatography column, and the protein SHG19-VP2-466 was eluted with buffer B (20 mM phosphate, 150 mM NaCl, pH 6.5). Finally, the purified protein samples were analyzed by sodium dodecyl sulfate polyacrylamide gel electrophoresis (SDS-PAGE) and Coomassie brilliant blue staining.

### 2.5. Analysis of SDS-PAGE and Western-Blot

Protein samples were mixed with a loading buffer (50 mM Tris/HCl, pH 6.8, 2% SDS, 5% *v/v* 2-mercaptoethanol, 10% *w/v* sucrose, 0.02% Bromophenol Blue), denatured at 100 °C for 10 min, subjected to SDS-PAGE and stained with Coomassie Brilliant Blue. For Western blotting, proteins in the SDS-PAGE gel were transferred onto nitrocellulose membrane. After being blocked with 5% (*w/v*) skimmed milk in phosphate-buffered saline (PBS), the membranes were incubated with an anti-VP2 monoclonal antibody (7D4) [20] or an anti-Flag antibody (Sigma -Aldrich, St. Louis, MO, USA) (negative control), at room temperature for 1.5 h. Then the membranes were washed three times with PBST (PBS containing 0.1% Tween 20) followed by incubation with IRDye 800CW goat anti-mouse secondary antibody (diluted 1:20,000) at room temperature for 45 min. Finally, the membranes were washed three times with PBST and detected with a Licor ODYSSEY (Licor, Lincoln, NE, USA) instrument.

### 2.6. Transmission Electron Microscopy (TEM)

Purified protein sample was negative stained by 2% (*w/v*) aqueous uranyl acetate, followed by observation under a HITACHI H-7650 microscope. Digital images were processed using iTEM software (Olympus Soft Imaging System GmbH, Munster, Germany).

### 2.7. Agar-Gel Precipitation (AGP)

The reference antiserum and antigen of IBDV for AGP detection were purchased from Harbin Guosheng Biological Company (Harbin, China). The purified SHG19-VP2-466 protein and reference IBDV antigen were added into the surrounding wells of the agarose plate, and the center well was filled with the reference IBD antiserum. The plate was incubated at 37 °C for 36 h.

### 2.8. Preparation of SHG19-VLP Vaccine

The purified SHG19-VLP solution was filtered using a 0.22-μm filter for sterilization. Based on the AGP titer, the SHG19-VLP solution was diluted to 3 log_2_ and 1 log_2_ with sterile PBS. Antigens with different AGP titers were emulsified with oil adjuvant (1:2).

### 2.9. Evaluation of the Immune Effect of SHG19-VLP Vaccine Against nVarIBDV

Twenty 2-week-old SPF chickens were randomly divided into four groups (n = 5 each group). Chickens in groups 1 and 2 were injected with 200 μL PBS intramuscularly. Chickens in groups 3 and 4 were vaccinated intramuscularly with 200 μL SHG19-VLP vaccines containing antigen of 3 log_2_ and 1 log_2_ AGP titer, respectively. At 13 days post-vaccination (d p.v.), serum samples were obtained from the wing vein to detect the presence of virus-neutralization antibodies. At 14 d p.v., chickens in group 2, 3, and 4 were challenged with 10 CID_50_ (50% chicken infection dose) of the nVarIBDV SHG19 strain via the intranasal and optical routes. Group 2 was a sham group (non-vaccinated challenge control); Group 1 without challenge were used as negative control (NC). The chickens were monitored daily for clinical signs. All chickens were euthanized and necropsied at 7 days post-challenge (d p.c.). The weights of the body and bursa were determined to calculate the bursa/body weight (B/BW) ratios (B/BW ratio = bursal weight/body weight × 1,000). The mean values and standard deviations of the data obtained from five independent chicken samples were calculated. Additionally, the bursa tissues were fixed in 10% neutral-buffered formalin for histopathology examination.

### 2.10. Evaluation of Immune Effect of SHG19-VLP Vaccine Against vvIBDV

Fifteen 2-week-old SPF chickens were randomly divided into three groups (n = 5 each group). Chickens in group 1 and 2 were administered with 200 μL PBS intramuscularly. Chickens in group 3 were vaccinated intramuscularly with 200 μL SHG19-VLP vaccines containing antigen of 1 log_2_ AGP titer. At 13 d p.v., serum samples were obtained from the wing vein for the virus-neutralization antibody detection. At 14 d p.c., groups 2 and 3 were challenged with 10 CLD_50_ (50% chicken lethal dose) of the vvIBDV HLJ0504 strain via the intranasal and optical routes. Group 2 was a sham group (non-vaccinated and challenge control); group 1 without challenge were used as NC. The chickens were monitored daily for clinical signs. At 7 d p.c., all the remaining chickens were euthanized and necropsied according to the procedure stated in Section 2.9.

### 2.11. Virus-Neutralization Assay

The rGtVarVP2 strain and rGtHLJVP2 strains of IBDV were used to detect serum neutralization antibody against VP2 antigen of nVarIBDV and vvIBDV, respectively, via the virus neutralization assay. The serum samples collected at 13 d p.v. were filtered through 0.22-μm filters after being inactivated at 56 °C for 30 min. In a 96-well plate, successive two-fold dilutions of the serum were mixed with 200 TCID_50_ of the rGtVarVP2 or rGtHLJVP2 strain followed by incubation at 37 °C for 1 h, which were then added to the DF1 cells. The initial serum dilution was 1:4. After being cultured in a constant temperature incubator at 37 °C for 72 h, the cytopathic effects (CPEs) were observed.

### 2.12. Statistical Analyses

A one-way ANOVA was used to evaluate the statistical significance of the differences among the different groups. A *p*-value < 0.05 was considered statistically significant.

## 3. Results

### 3.1. Expression, Purification, and Identification of SHG19-VLP

To generate the fusion protein SHG19-VP2-466 (Figure 1a), the recombinant plasmid pCo-HHT28-SHGVP2-466 was transformed into competent *E. coli* Transetta (DE3) cells. The result of Western blotting showed that a 55-kDa band, corresponding to the molecular mass of the fusion protein SHG19-VP2-466, was detected by the anti-VP2 MAb. No specific band was detected in the pCold I (empty vector)-transformed *E. coli* Transetta (DE3) (Figure 1b). A non-specific antibody (anti-Flag) was used as a negative control for the Western blotting. The result showed that no specific band was observed in the pCo-HHT28-SHGVP2-466 and pCold I-transformed *E. coli* Transetta (DE3) (Figure 1c). The result of SDS-PAGE showed that after ammonium sulfate precipitation and size-exclusion chromatography, the fusion protein SHG19-VP2-466 was purified successfully (Figure 1d). The purified protein was then examined via TEM, and VLPs with a diameter of about 25 nm were observed (Figure 1e). The AGP assay showed that the titer of SHG19-VLP reached 4 log_2_ (Figure 1f).

### 3.2. SHG19-VLP Vaccine Provided Protection against nVarIBDV Challenge

**Figure 2:**
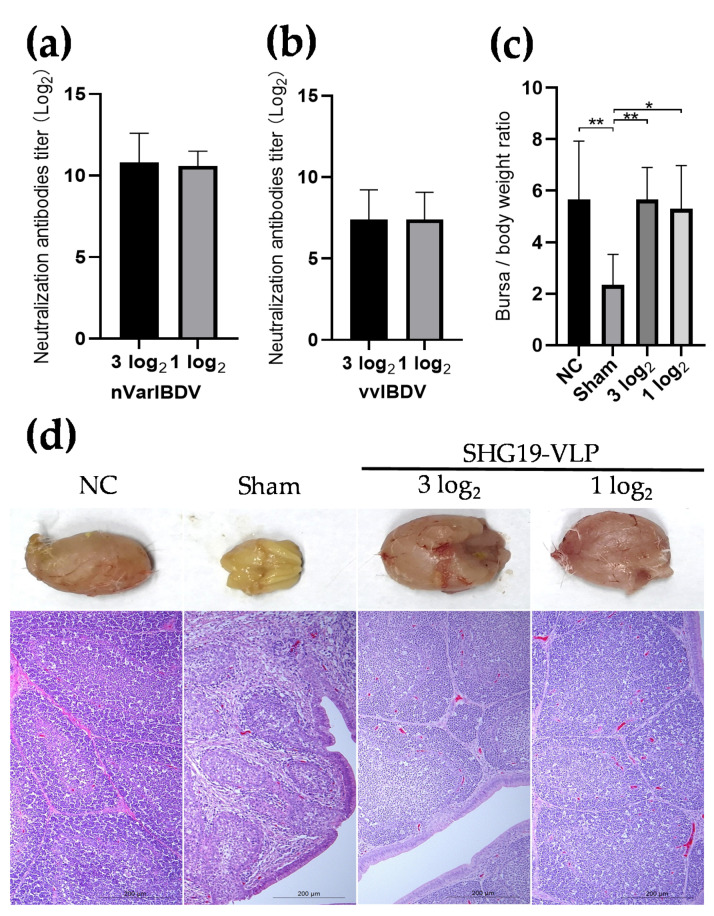
Evaluation of the immune effect of the SHG19-VLP vaccine against nVarIBDV. (**a**) Serum neutralization antibody titers against nVarIBDV antigen (rGtVarVP2) at 13 days post-vaccination; (**b**) Serum neutralization antibody titers against vvIBDV (rGtHLJVP2) at 13 days post-vaccination; (**c**) B/BW ratio at 7 days post-challenge. The average values and standard deviations (error bars) from different independent samples are shown; Asterisk signs were used to determine statistical significance among different groups (* *p* < 0.05; ** *p* < 0.01); (**d**) Gross appearance (upper side) and corresponding histopathological appearance (lower side) of the bursal sections (hematoxylin and eosin staining) at 7 days post-challenge.

Two-week-old SPF chickens were vaccinated with SHG19-VLP at two different doses to evaluate the immunogenicity and protective efficacy of the SHG19-VLP vaccine against nVarIBDV. The sera of all chickens were collected at 13 d p.v., and the antibody titers were measured via a virus-neutralization assay. The results showed that both vaccinated groups were positive for the nVarIBDV-specific neutralization antibody with titers of 10.80 ± 1.79 log_2_ and 10.60 ± 0.89 log_2_, respectively (Figure 2a). In addition, SHG19-VLP vaccination also induced vvIBDV-specific neutralization antibody at titers of 7.40 ± 1.82 log_2_ and 7.40 ± 1.67 log_2_, respectively (Figure 2b), which were comparatively lower than that of the nVarIBDV-specific neutralization antibody. The serum neutralization antibody values were below 2 log_2_ in two non-vaccinated groups.

In a subsequent challenge experiment using the nVarIBDV SHG19 strain, no clinical symptoms were observed in either vaccinated group. At 7 d p.c., the chickens were euthanized for necropsy. Compared with the NC group, the bursae of chickens in the non-vaccinated challenge control group (sham group) were significantly atrophied, and turned yellow with inflammatory exudation. Comparatively, no gross lesions were observed in the bursae of chickens in both vaccinated groups (3 log_2_ and 1 log_2_) (Figure 2d). The mean B/BW ratio of non-vaccinated challenge control group (sham group) was significantly lower than that of the NC group, while the mean B/BW ratios of both vaccinated groups were not significantly different from that of the NC group (Figure 2c). The results of histopathology examination further revealed obvious histopathological lesions in the bursae of the non-vaccinated challenge control group, including follicle atrophy, interstitial hyperplasia, and lymphopenia. However, similar to the bursae in the NC group, no microscopic lesions were observed in either SHG19-VLP vaccinated groups after challenge (Figure 2d).

### 3.3. SHG19-VLP Vaccine Induced Protection against vvIBDV Infection

**Figure 3:**
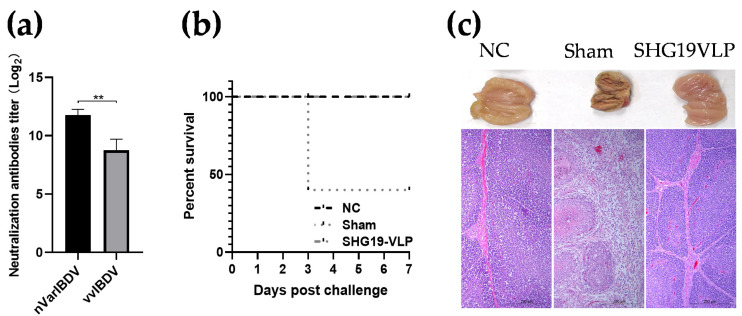
Evaluation of the immune effect of the SHG19-VLP vaccine against vvIBDV. (**a**) Serum neutralization antibody titers at 13 days post-vaccination. The average values and standard deviations (error bars) from different independent samples are shown; Asterisk sign was used to determine statistical significance among different groups (** *p* < 0.01); (**b**) Survival curve. (**c**) The gross appearance (upper side) and corresponding histopathological appearance (lower side) of the bursal sections (hematoxylin and eosin staining) at 7 days post-challenge.

To further evaluate the immune protection efficacy of the SHG19-VLP vaccine against vvIBDV, another animal experiment was performed. In this experiment, 2-week-old SPF chickens were vaccinated with the SHG19-VLP vaccine (1 log_2_ AGP titer per chicken). At 13 d p.v., the SHG19-VLP vaccine induced neutralization antibodies not only against nVarIBDV (11.75 ± 0.50 log_2_) but also vvIBDV (8.75 ± 0.48 log_2_) (Figure 3a). The serum neutralization antibody values were below 2 log_2_ in the two non-vaccinated groups. At 14 d p.v., the other two groups were challenged with the vvIBDV HLJ0504 strain, except the NC group. In the non-vaccinated challenge control group (sham group), HLJ0504 caused 100% (5/5) morbidity and 60% mortality (3/5) while no obvious clinical symptoms were observed in the vaccinated group and the NC group (Figure 3b). At 7 d p.c., compared with the NC group, the bursae of two survival chickens in the non-vaccinated challenge control group (sham group) were significantly atrophied with severe pathological lesions including follicle atrophy, interstitial hyperplasia, and lymphopenia. No gross and microscopic lesions were observed in the vaccinated group (Figure 3c).

## 4. Discussion

Recently, the nVarIBDV epizootic in East Asia including China [20] and Japan [23], has posed serious challenges for the prevention and control of IBD. The nVarIBDV has caused enormous economic losses for its ability to destroy the bursae of infected chickens, causing severe immunosuppression, interfering with the protective efficacy of some avian vaccines (including Newcastle disease vaccine and avian influenza vaccine), and seriously affecting the weight gain of infected chickens [20,21]. Due to the antigenicity mismatch, some vvIBDV vaccines such as attenuated vaccine [27], viral-like particle vaccine [25], and combined vaccine [22] could not provide 100% protection against bursal lesion caused by nVarIBDV [22,25], resulting in the continuous spread of nVarIBDV in immunized flocks [22,25], which has become a huge threat to the development of poultry industry. Therefore, it is necessary and urgent to develop newly effective preventative vaccine against nVarIBDV.

Live and inactivated vaccines are widely available for the control of IBD. However, these vaccines have the potential risks of incomplete inactivation and reversion to virulence [30]. A recent study reported that the homologous recombination between the nVarIBDV and IBDV intermediate vaccine strain increased the pathogenicity of nVarIBDV [31]. Subunit vaccines that do not contain complete viral particles and viral nucleic acid components have become a new direction for the development of vaccines. VP2, the main host-protective antigen of IBDV, is usually used as an immunogen of subunit vaccines to elicit a protective immune response to IBDV [8,32]. Several expression systems, including yeast [33,34], insect cells [35], mammalian cells [29], plant cells [36,37], and *E. coli* [38] have been used to produce the VP2 of IBDV. Eukaryotic expression systems produce highly bioactive recombinant proteins, but their application is limited by various technical problems, such as low yield and high cost. Compared with eukaryotic expression systems, *E. coli* is more suitable to produce avian vaccines owing to its advantages of high yield, low cost, ease of manipulate and scale-up. So it was widely used as a cell factory to produce vaccines [39,40]. In this study, the recombinant protein SHG19-VP2-466 was expressed successfully using *E. coli*, and it was purified via ammonium sulfate precipitation and size-exclusion chromatography. The C-terminal domain (aa 442-512) of pVP2 (512 residues) has an important role in determining the various conformations of VP2 (441 residues, aa 1-441 of pVP2) that build the capsid [41]. C-terminal domain of pVP2 contains four amphipathic α-helix which referred to as helix α5 (aa 433-452), α6 (aa 456-462), α7 (aa 468-471), and α8 (aa 476-481) [41]. Among them, helix α5 is the conformational switch of the VP2 polymorphism. Expression of mature VP2 alone results in the spontaneous assembly of VLPs with a diameter of approximately 23 nm. pVP2 or intermediate pVP2 variant expression leads to tubular structures [42]. Electrostatic interactions between C-terminal of VP3 and helix α5 are essential for the correct assembly of VP2 [42]. It has also been reported that a special His-tag can emulate the role of C-terminal of VP3 in contributing to the production of VLP [29,42]. In the design of recombinant plasmids in this study, the α5-helix and α6-helix at the C-terminus of pVP2 were retained, and the special His-tag sequence was fused at the N-terminal of VP2. TEM result revealed that the purified VP2 protein efficiently assembled into VLPs with a diameter of approximately 25 nm, which was similar to IBDV virions [42]. The SHG19-VLP was stable and could retain a typical morphological appearance for at least 12 months at 4 °C (data not shown).

Humoral immunity is essential for the immune protection of IBDV [15]. The serum neutralizing antibody titer is an important indicator to evaluate the strength of the humoral immune response. Similar to other wild-type IBDV, nVarIBDV cannot adapt in vitro in cell line such as DF1 cell, so detection of the neutralizing antibody against nVarIBDV is inconvenient. We previously constructed a recombinant IBDV named rGtVarVP2 which not only expressed the protective antigen VP2 of nVarIBDV but also induced CPE in DF1 cells [27], which enabled us to easily evaluate the serum neutralizing antibody titer against nVarIBDV antigen according to the CPE. In addition, another DF1-adapted IBDV strain named rGtHLJVP2, which expressed the protective antigen VP2 of vvIBDV, was also previously rescued [28]. This virus can be used to evaluate the neutralizing antibody titer against vvIBDV antigen. The results of virus-neutralization assay showed that even if chickens were immunized once with 1 log_2_ AGP titer, the SHG19-VLP vaccine could efficiently induce efficiently neutralization antibody (10.6 ± 0.9 log_2_), providing 100% protection against nVarIBDV.

In another recent publication [25], a nVarIBDV-VLP with the diameter of 14–17 nm was developed. Such a diameter of IBDV-VLP has not been reported. This VLP could provide good protection against nVarIBDV, but its protection efficiency against vvIBDV has not been evaluated. The results of the first animal experiment of our study also showed that the SHG19-VLP could elicit vvIBDV-specific neutralization antibodies. To confirm this, the second animal experiment was performed. Induction of the vvIBDV-specific neutralization antibody by the SHG19-VLP vaccine was confirmed although the titer was about 3 log_2_ lower than that of the nVarIBDV-specific neutralization antibody. The challenge experiment further showed that SHG19-VLP vaccine also provided 100% immune protection against the lethal challenge of vvIBDV.

A previous study suggested that classic strain vaccine could fully protect against the classic strain but only provide partial protection against variant IBDV [43], while variant strain-vaccine could protect against both themselves and the classic strain of IBDV [44]. The vvIBDV shares similar antigen with classic strain of IBDV [45]. Our recently published data showed that nVarIBDV-specific neutralization antibody titers induced by a commercial vvIBDV vaccine (B87 strain) was obviously lower than that induced by a nVarIBDV vaccine candidate strain. And the B87 vaccine only provided 50% (5/10) protection against the novel variant IBDV [27]. In addition, a recent study also confirmed that a subunit vaccine against vvIBDV could not provide complete protection against nVarIBDV [25]. Failure of vaccination is related mainly to antigenic variants [45,46]. Several amino acids have been identified to be closely associated to the antigenicity of IBDV [47]. Why could the variant strain-vaccine provide broad-spectrum protection? This phenomenon might be related to the number of universal neutralizing epitopes on VP2. As showed in the supplementary Table S1 and the data of the relative publication [20], compared with vvIBDV and cIBDV, 13 characteristic aa residues of 213N, 222T, 242V, 249K, 256V, 253Q, 279N, 284A, 286I, 294L, 318D, 323E, and 330S, were observed in the hypervariable region (HVR) of VP2 of variant IBDV. Among variant IBDV, nVarIBDV showed three distinct aa residues in HVR, including 221K, 252I, and 299S. These aa differences might be involved in antigen epitopes variation. Further studies on the universal epitopes of different subtypes of IBDV will not only contribute to understanding the mechanisms of IBDV antigenic variation, but may also provide new ideas for the development of broad-spectrum IBDV vaccines.

Usually, to control various poultry infectious disease, chickens have to be administered many vaccinations, which would inevitably cause different levels of side effects. A combination vaccine can reduce the immunization times and improve animal welfare as it can prevent multiple diseases by single shot. Therefore, it is also valuable and convenient to use SHG19-VLP as the component of a combination vaccine. Immunizing chickens at 1-day-old or in ovo are convenient ways of immunization. In future studies, we will evaluate the immune protective efficacy of the SHG19-VLP vaccine at different immune ages, and explore the optimal immune procedure in the presence of maternal antibodies.

## 5. Conclusions

The nVarIBDV-VP2, which can self-assemble into VLP, was successfully expressed using an *E. coli* expression system. The SHG19-VLP candidate vaccine can provide complete immune protection not only against the homologous nVarIBDV but also the heterologous vvIBDV. This research is of great significance to the comprehensive prevention and control of the most recent epidemic of atypical IBD and the further successful development of the poultry industry.

## Data Availability

Data can be requested by writing to the author.

## Supplementary Materials

The following are available online at https://www.mdpi.com/2076-393X/9/2/142/s1, Table S1: Characteristic amino acid substitutions in the hypervariable region of VP2. nVarIBDV, novel variant strain; VarIBDV, variant strain; vvIBDV, very virulent strain; cIBDV, classic strain; An asterisk indicates a residue identical to the SHG19 strain sequence.

## References

[1] Müller H., Islam M.R., Raue R. (2003). Research on infectious bursal disease—the past, the present and the future. Vet. Microbiol..

[2] Brown M.D., Skinner M.A. (1996). Coding sequences of both genome segments of a European ‘very virulent’ infectious bursal disease virus. Virus Res..

[3] Lombardo E., Maraver A., Espinosa I., Fernandez-Arias A., Rodriguez J.F. (2000). VP5, the nonstructural polypeptide of infectious bursal disease virus, accumulates within the host plasma membrane and induces cell lysis. Virology.

[4] Petit S., Lejal N., Huet J.C., Delmas B. (2000). Active residues and viral substrate cleavage sites of the protease of the birnavirus infectious pancreatic necrosis virus. J. Virol..

[5] Sánchez A.B., Rodriguez J.F. (1999). Proteolytic processing in infectious bursal disease virus: Identification of the polyprotein cleavage sites by site-directed mutagenesis. Virology.

[6] Chevalier C., Lepault J., Erk I., Da Costa B., Delmas B. (2002). The maturation process of pVP2 requires assembly of infectious bursal disease virus capsids. J. Virol..

[7] Dey S., Pathak D.C., Ramamurthy N., Maity H.K., Chellappa M.M. (2019). Infectious bursal disease virus in chickens: Prevalence, impact, and management strategies. Vet. Med..

[8] Jackwood D.J. (2017). Advances in vaccine research against economically important viral diseases of food animals: Infectious bursal disease virus. Vet. Microbiol..

[9] Letzel T., Coulibaly F., Rey F.A., Delmas B., Jagt E., Van Loon A.A., Mundt E. (2007). Molecular and structural bases for the antigenicity of VP2 of infectious bursal disease virus. J. Virol..

[10] Brandt M., Yao K., Liu M., Heckert R.A., Vakharia V.N. (2001). Molecular determinants of virulence, cell tropism, and pathogenic phenotype of infectious bursal disease virus. J. Virol..

[11] Jackwood D.J., Sreedevi B., LeFever L.J., Sommer-Wagner S.E. (2008). Studies on naturally occurring infectious bursal disease viruses suggest that a single amino acid substitution at position 253 in VP2 increases pathogenicity. Virology.

[12] Ye C., Wang Y., Zhang E., Han X., Yu Z., Liu H. (2018). VP1 and VP3 are required and sufficient for translation initiation of uncapped infectious bursal disease virus genomic double-stranded RNA. J. Virol..

[13] Wu H., Shi L., Zhang Y., Peng X., Zheng T., Li Y., Hu B., Zheng X., Zhou J. (2019). Ubiquitination is essential for Avibirnavirus replication by supporting VP1 polymerase activity. J. Virol..

[14] Lombardo E., Maraver A., Castón J.R., Rivera J., Fernández-Arias A., Serrano A., Carrascosa J.L., Rodriguez J.F. (1999). VP1, the putative RNA-dependent RNA polymerase of infectious bursal disease virus, forms complexes with the capsid protein VP3, leading to efficient encapsidation into virus-like particles. J. Virol..

[15] Yang H., Wang Y., Ye C. (2020). Rapid generation of attenuated infectious bursal disease virus from dual-promoter plasmids by reduction of viral ribonucleoprotein activity. J. Virol..

[16] Cosgrove A.S. (1962). An apparently new disease of chickens: Avian nephrosis. Avian Dis..

[17] Jackwood D.H., Saif Y.M. (1987). Antigenic diversity of infectious bursal disease viruses. Avian Dis..

[18] Chettle N., Stuart J.C., Wyeth P.J. (1989). Outbreak of virulent infectious bursal disease in East Anglia. Vet. Rec..

[19] Prabhu S.N., Singh A.P., Varghese B.P., Dhama K., Singh S.D., Singh R. (2020). A comparative study of pathology and host immune response induced by very virulent infectious bursal disease virus in experimentally infected chickens of Aseel and white Leghorn breeds. Vaccines.

[20] Fan L., Wu T., Hussain A., Gao Y., Zeng X., Wang Y., Gao L., Li K., Wang Y., Liu C. (2019). Novel variant strains of infectious bursal disease virus isolated in China. Vet. Microbiol..

[21] Xu A., Pei Y., Zhang K., Xue J., Ruan S., Zhang G. (2019). Phylogenetic analyses and pathogenicity of a variant infectious bursal disease virus strain isolated in China. Virus Res..

[22] Fan L., Wu T., Wang Y., Hussain A., Jiang N., Gao L., Li K., Gao Y., Liu C., Cui H. (2020). Novel variants of infectious bursal disease virus can severely damage the bursa of fabricius of immunized chickens. Vet. Microbiol..

[23] Myint O., Suwanruengsri M., Araki K., Izzati U.Z., Pornthummawat A., Nueangphuet P., Fuke N., Hirai T., Jackwood D.J., Yamaguchi R. (2020). The bursa atrophy at 28 days old by the variant infectious bursal disease virus makes a negative economic impact on broiler farms in Japan. Avian Pathol..

[24] Muller H., Mundt E., Eterradossi N., Islam M.R. (2012). Current status of vaccines against infectious bursal disease. Avian Pathol..

[25] Li G., Kuang H., Guo H., Cai L., Chu D., Wang X., Hu J., Rong J. (2020). Development of a recombinant VP2 vaccine for the prevention of novel variant strains of infectious bursal disease virus. Avian Pathol..

[26] Qi X., Gao L., Qin L., Deng X., Wu G., Zhang L., Yu F., Ren X., Gao Y., Gao H. (2011). Genomic sequencing and molecular characteristics of a very virulent strain of infectious bursal disease virus isolated in China. Agric. Sci. Technol..

[27] Fan L., Wang Y., Jiang N., Gao L., Li K., Gao Y., Cui H., Pan Q., Liu C., Zhang Y. (2020). A reassortment vaccine candidate of the novel variant infectious bursal disease virus. Vet. Microbiol..

[28] Gao L., Qi X., Li K., Gao H., Gao Y., Qin L., Wang Y., Wang X. (2011). Development of a tailored vaccine against challenge with very virulent infectious bursal disease virus of chickens using reverse genetics. Vaccine.

[29] Saugar I., Irigoyen N., Luque D., Carrascosa J.L., Rodríguez J.F., Castón J.R. (2010). Electrostatic interactions between capsid and scaffolding proteins mediate the structural polymorphism of a double-stranded RNA virus. J. Biol. Chem..

[30] Dey S., Chellappa M.M., Pathak D.C., Gaikwad S., Yadav K., Ramakrishnan S., Vakharia V.N. (2017). Newcastle disease virus vectored bivalent vaccine against virulent infectious bursal disease and Newcastle disease of chickens. Vaccines.

[31] Wu T., Wang Y., Li H., Fan L., Jiang N., Gao L., Li K., Gao Y., Liu C., Cui H. (2020). Naturally occurring homologous recombination between novel variant infectious bursal disease virus and intermediate vaccine strain. Vet. Microbiol..

[32] Huo S., Zhang J., Fan J., Wang X., Wu F., Zuo Y., Zhong F. (2019). Co-expression of chicken IL-2 and IL-7 enhances the immunogenicity and protective efficacy of a VP2-eexpressing DNA vaccine against IBDV in chickens. Viruses.

[33] Wang M., Pan Q., Lu Z., Li K., Gao H., Qi X., Gao Y., Wang X. (2016). An optimized, highly efficient, self-assembled, subvirus-like particle of infectious bursal disease virus (IBDV). Vaccine.

[34] Arnold M., Durairaj V., Mundt E., Schulze K., Breunig K.D., Behrens S.E. (2012). Protective vaccination against infectious bursal disease virus with whole recombinant Kluyveromyces lactis yeast expressing the viral VP2 subunit. PLoS ONE.

[35] Jackwood D.J. (2013). Multivalent virus-like–particle vaccine protects against classic and variant infectious bursal disease viruses. Avian Dis..

[36] Lucero M.S., Richetta M., Chimeno Zoth S., Jaton J., Pinto S., Canet Z., Berinstein A., Gómez E. (2019). Plant-based vaccine candidate against Infectious bursal disease: An alternative to inactivated vaccines for breeder hens. Vaccine.

[37] Rage E., Drissi Touzani C., Marusic C., Lico C., Göbel T., Bortolami A., Bonfante F., Salzano A.M., Scaloni A., Fellahi S. (2019). Functional characterization of a plant-produced infectious bursal disease virus antigen fused to the constant region of avian IgY immunoglobulins. Appl. Microbiol. Biotechnol..

[38] Jiang D., Liu Y., Wang A., Zhang G., Yang G., Chen Y., Ji P., Liu C., Song Y., Su Y. (2016). High level soluble expression and one-step purification of IBDV VP2 protein in Escherichia coli. Biotechnol. Lett..

[39] Mustafa S., Abd-Aziz N., Saw W.T., Liew S.Y., Yusoff K., Shafee N. (2020). Recombinant enterovirus 71 viral protein 1 fused to a truncated Newcastle disease virus NP (NPt) carrier protein. Vaccines.

[40] Feodorova V.A., Lyapina A.M., Zaitsev S.S., Khizhnyakova M.A., Sayapina L.V., Ulianova O.V., Ulyanov S.S., Motin V.L. (2019). New promising targets for synthetic Omptin-based peptide vaccine against gram-negative pathogens. Vaccines.

[41] Irigoyen N., Castón J.R., Rodríguez J.F. (2012). Host proteolytic activity is necessary for infectious bursal disease virus capsid protein assembly. J. Biol. Chem..

[42] Saugar I., Luque D., Ona A., Rodriguez J.F., Carrascosa J.L., Trus B.L., Caston J.R. (2005). Structural polymorphism of the major capsid protein of a double-stranded RNA virus: An amphipathic alpha helix as a molecular switch. Structure.

[43] Eterradossi N., Saif Y.M., Swayne D.E., Boulianne M., Logue C.M., McDougald L.R., Nair V., Suarez D.L., Wit S., Grimes T., Johnson D., Kromm M. (2020). Infectious Bursal Disease. Diseases of Poultry.

[44] Ismail N.M., Saif Y.M. (1991). Immunogenicity of infectious bursal disease viruses in chickens. Avian Dis..

[45] He X., Wang W., Chen G., Jiao P., Ji Z., Yang L., Wei P. (2019). Serological study reveal different antigenic IBDV strains prevalent in southern China during the years 2000–2017 and also the antigenic differences between the field strains and the commonly used vaccine strains. Vet. Microbiol..

[46] Le X.T.K., Doan H.T.T., Do R.T., Le T.H. (2019). Molecular characterization of field isolates of infectious bursal disease virus from three decades, 1987-2018, reveals a distinct genotypic subgroup in Vietnam. Arch. Virol..

[47] Boudaoud A., Mamache B., Tombari W., Ghram A. (2016). Virus mutations and their impact on vaccination against infectious bursal disease (Gumboro disease). Rev. Sci. Tech..

