# Supplementary material for: Development of a Viral-Like Particle Candidate Vaccine Against Novel Variant Infectious Bursal Disease Virus
Source: Vaccines (Basel). 2021 Feb 10;9(2):142. doi: 10.3390/vaccines9020142 (PMC7916800; doi:10.3390/vaccines9020142)
Supplement: Supplementary file 1 [file vaccines-09-00142-s001.pdf]

Strain	phenotype	GenBank No.	VP2																
			213	221	222	242	249	252	253	254	256	279	284	286	294	299	318	323	330
SHG19	nVarIBDV	MH879092	N	K	T	V	K	I	Q	N	V	N	A	I	L	S	D	E	S
SHG352	nVarIBDV	MT179720	*	*	*	*	*	*	*	*	*	*	*	*	*	*	*	*	*
Variant E USA	VarIBDV	AF133904	*	Q	*	*	*	V	*	S	*	*	*	*	*	N	*	*	*
9109 USA	VarIBDV	AY462027	*	Q	*	*	*	V	*	*	*	*	*	*	*	N	N	D	*
Variant A USA	VarIBDV	M64285	D	Q	Q	*	*	V	*	S	*	*	*	*	*	N	*	D	*
GLS USA	VarIBDV	AY368653	D	Q	*	*	*	V	*	S	*	*	*	T	*	N	G	D	*
BD399 Bangladesh	vvIBDV	AF362776	D	Q	A	I	Q	V	*	G	I	D	*	T	I	*	G	D	*
HK46 China	vvIBDV	AF092943	D	Q	A	I	Q	V	*	G	I	D	*	T	I	*	G	D	*
UK661 France	vvIBDV	NC-004178	D	Q	A	I	Q	V	*	G	I	D	*	T	I	*	G	D	*
D6948 Netherlands	vvIBDV	AF240686	D	Q	A	I	Q	V	*	G	I	D	*	T	I	*	G	D	*
YS07 China	vvIBDV	FJ695138	D	Q	A	I	Q	V	*	G	I	D	*	T	I	*	G	D	*
OKYM Japan	vvIBDV	D49706	D	Q	A	I	Q	V	*	G	I	D	*	T	I	*	G	D	*
IM USA	cIBDV	AY029166	D	Q	P	I	Q	V	*	G	*	D	*	T	*	N	G	D	*
F52/70 France	cIBDV	HG974565	D	Q	P	I	Q	V	*	G	*	*	*	T	*	N	G	D	*
